# Steuerpolitik in der COVID-19-Krise

**DOI:** 10.1007/s10273-020-2761-9

**Published:** 2020-10-24

**Authors:** Sebastian Eichfelder, Jonathan Hoke

**Affiliations:** Lehrstuhls für Betriebswirtschaftliche Steuerlehre, Otto-von-Guericke-Universität Magdeburg, Universitätsplatz 2, 39106 Magdeburg, Deutschland

**Keywords:** H25, H71

## Abstract

Die Corona-Krise hat die Debatte über eine steuerliche Entlastung von Unternehmen neu belebt. Tatsächlich haben Reformen in Deutschland bereits seit 20 Jahren die Unternehmensbesteuerung deutlich gesenkt. Ziel ist es, im internationalen Steuerwettbewerb zu bestehen. Allerdings geht die neue ökonomische Geografie davon aus, dass es sich Volkswirtschaften mit hoher Standortattraktivität leisten können, hohe Steuern zu erheben. Für die Standortwahl der Unternehmen sind stabile wirtschaftliche Rahmenbedingungen, eine ausgebaute Infrastruktur und gut ausgebildete Arbeitskräfte wesentlich wichtiger. Die Steuerpolitik in der COVID-19-Pandemie sollte sich dementsprechend darauf konzentrieren, diese Standortfaktoren zu verbessern.

## Abstract (en)

Current reform proposals call for a reduction of the corporate tax burden in Germany and justify this due to the competitiveness of Germany as a business location. However, in view of Germany’s continuously high attractiveness in this regard, these demands appear to be inappropriate, even in an international context. To ensure this attractiveness in the long term, it seems to be more important to focus on investments in digitisation, the digital infrastructure, and electromobility. Even in times of economic problems, e.g., the COVID19 pandemic, lowering the nominal tax burdens on companies is not necessarily the answer. Instead, temporary tax breaks for companies that invest during this phase of economic weakness may be more appropriate.

Infolge der US-amerikanischen Steuerreform 2017 sowie konjunktureller Probleme im Zuge der COVID-19-Pandemie mehren sich in Deutschland Stimmen, die eine strukturelle Reform der Unternehmensbesteuerung anmahnen (etwa Fuest und Peichl, 2020; Homburg, 2020; Hüther, 2020; Welling, 2018; Wissenschaftlicher Beirat beim Bundesministerium der Finanzen, 2018; Wolff, 2018). So wird argumentiert, die Steuerreform der USA könne Investitionsentscheidungen von Unternehmen erheblich beeinflussen (Heinemann et al., 2017, 3). Im Koalitionsvertrag 2018 plante die Große Koalition, den Steuerwettbewerb zwischen den EU-Staaten zu unterbinden und eine „europäische Antwort auf internationale Veränderungen und Herausforderungen [bei den Unternehmensteuern], nicht zuletzt in den USA, zu geben" (CDU et al., 2018, 7-8, 13).

Diese geplanten Veränderungen wurden konkretisiert, indem vier Kernelemente einer möglichen Unternehmensteuerreform durch Bundeswirtschaftsminister Peter Altmaier (CDU) genannt wurden: 1. Verbesserungen und Entlastungen bei der Thesaurierungsbegünstigung für Personenunternehmen sowie ergänzend die Einführung eines Optionsmodells zur Körperschaftsbesteuerung; 2. Verbesserung und Erhöhung der Gewerbesteueranrechnung auf die Einkommensteuer sowie Einführung der Anrechenbarkeit der Gewerbesteuer auf die Körperschaftsteuer; 3. moderate Absenkung des Körperschaftsteuersatzes; 4. schrittweise Abschaffung der gewerbesteuerlichen Hinzurechnungen (Bundesministerium für Wirtschaft und Energie, 2019; CDU/CSU-Fraktion, 2019). Auch die vom bayerischen Ministerpräsidenten Markus Söder jüngst wieder ins Gespräch gebrachten Reformvorschläge (Börse Online, 2020) gehen in eine ähnliche Richtung. Kern aller dieser Vorschläge ist das Bestreben, die steuerliche Belastung von Unternehmen und insbesondere von Kapitalgesellschaften zu senken, um im internationalen Standortwettbewerb mithalten zu können. Auch wenn die bisherigen Reformpakete der Bundesregierung keine erheblichen Entlastungen bei den Unternehmensteuern vorsehen (Beck Online, 2020a, 2020b; Korn, 2020), ist anzunehmen, dass die Diskussion um eine nachhaltige Senkung der Steuerbelastung von Unternehmen künftig wieder Fahrt aufnehmen wird.

Diese Diskussion knüpft an eine Entwicklung an, die bereits mit dem Auslaufen der Vermögensteuer im Jahr 1997 sowie den Reformgesetzen der rot-grünen Bundesregierung von 1999 bis 2002 ihren Anfang genommen hat und sich in der Unternehmensteuerreform 2008/2009 fortsetzte (Bach, 2018). Lag die nominale Steuerbelastung auf thesaurierte Gewinne von Kapitalgesellschaften bei einem gewerbesteuerlichen Hebesatz von 400 % und unter Vernachlässigung der Kirchensteuer im Jahr 1998 noch bei 56,2 %, beträgt sie heute 29,9 %. Bei ausgeschütteten Gewinnen von Kapitalgesellschaften ergibt sich eine Senkung der nominalen Steuerbelastung von 63,3 % auf 48,3 %, während sich die Spitzenbelastung von Personengesellschaften ebenfalls deutlich von 58,0 % auf 47,5 % vermindert hat (eigene Berechnungen). Berücksichtigt man weiterhin die Abschaf-

**Jonathan Hoke** ist dort studentischer Mitarbeiter.

fung der Gewerbekapitalsteuer, die faktische Abschaffung der Vermögensteuer, die Einführung der Abgeltungsteuer im Jahr 2009 sowie die massiven Steuervergünstigungen für Erbschaften von Unternehmenvermögen, dann wird deutlich, dass insbesondere Unternehmen und deren Anteilseigner als Gewinner der steuerlichen Reformpolitik der letzten 20 Jahre gelten können.

Aus dieser Perspektive erscheinen die aktuell diskutierten Reformvorschläge nicht unbedingt sozial ausgewogen. Die angemahnte strukturelle Reform des deutschen Steuersystems bedeutet insbesondere auch eine Umverteilung der Steuerbelastung auf Arbeitseinkommen und Konsumausgaben, während sich die Belastung von Unternehmengewinnen und Kapitaleinkommen erheblich reduziert. Es stellt sich die Frage, ob eine derart ausgerichtete Steuerpolitik noch dem Grundgedanken der Besteuerung nach der Leistungsfähigkeit entspricht. Es erscheint zudem bedenklich, dass eine derartige Politik die im öffentlichen Diskurs monierte Spaltung der Gesellschaft in Arm und Reich negiert und womöglich weiter verstärkt (Bartels, 2018).

## Steuerwettbewerb und neue ökonomische Geografie

Es ist grundsätzlich unstrittig, dass der Wettbewerbsdruck im Bereich der Unternehmensbesteuerung in jüngster Zeit zugenommen hat. Die USA haben mit dem „Tax Cuts and Jobs Act" zum 1.1.2018 die Federal Income Tax von 35 % auf 21 % gesenkt. In den OECD-Staaten fiel die durchschnittliche Unternehmensbesteuerung von etwa 32 % auf 24 % von 2000 bis 2019. So liegt auch die durchschnittliche Steuerbelastung der Unternehmen in der EU bei etwa 21 % (OECD, 2019). Die Unternehmensteuern sind in den vergangenen Jahren unter anderem in Belgien (auf 25 %), Großbritannien (auf 19 %), Japan (auf 29,7 %) und Italien (auf 27,8 %) erheblich gesenkt worden. Weitere Steuersenkungen sind geplant in Frankreich, Griechenland, Niederlande, Norwegen und Schweden (vgl. hierzu auch Abbildung 1). Deutschland wird sich dem daraus resultierenden Wettbewerbsdruck nicht vollständig entziehen können (Schreiber et al., 2018, 242). Allerdings stellt sich die Frage, welcher Stellenwert der Besteuerung bei Standortwahl- und Investitionsentscheidungen grundsätzlich zukommt.

Befragungen von Unternehmen kommen üblicherweise zu dem Ergebnis, dass Steuern zwar einen wesentlichen aber beileibe nicht den wichtigsten Standortfaktor darstellen. Nach einer Metastudie des Ifo-Instituts Dresden werden Steuern und Abgaben als fünftwichtigster Faktor für die Standortwahl zwischen verschiedenen Kommunen eingeordnet (Ebertz et al., 2008, 18). Größere Bedeutung haben das Angebot an qualifizierten Arbeitskräften, die Kundennähe, das Lohnniveau und die Verkehrsanbindung/Infrastruktur. Eine Studie der Kreditanstalt für Wiederaufbau, die deutlich mehr harte und weiche Standortfaktoren berücksichtigt, steht die Besteuerung sogar eher im unteren Mittelfeld der relevanten Standortfaktoren (Landua et al., 2017). Der von der Weltbank jährlich veröffentlichte Ease-of-Doing-Business-Index platziert Deutschland im Jahr 2020 auf Rang 22, wobei Steuern auch hier nur einer von vielen Faktoren sind (Linnemann und Weiß, 2019; Weltbank, 2020). Internationale Untersuchungen, die mehrere Standortfaktoren vergleichen, kommen zu ähnlichen Ergebnissen (etwa Kimelberg und Williams, 2013; MacCarthy and Atthirawong, 2003; Vlachou und Iakovidou, 2015). Während die rein steuerliche Literatur einen signifikanten Einfluss der Besteuerung auf Standortwahlentscheidungen und insbesondere Direktinvestitionen feststellen kann (Feld und Heckemeyer, 2011; Hebous et al., 2011), sollte ihre Bedeutung auf die Standortwahl internationaler Unternehmen nicht überschätzt werden.

Abbildung 1
**Entwicklung der Steuersätze auf Kapitalgesellschaften in OECD-Staaten**

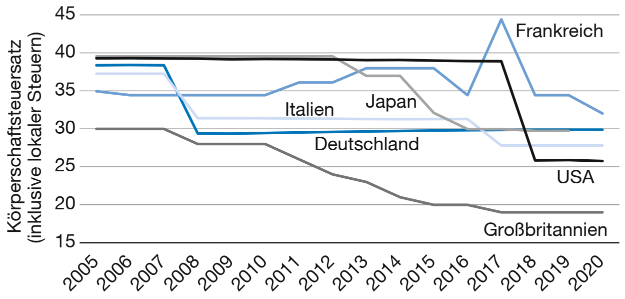

Quelle: OECD.Stat, 2020.

Eine theoretische Grundlage für die Relevanz der Unternehmensbesteuerung für die Standortwahl bietet die ökonomische Geografie für die Paul Krugman im Jahr 2008 mit dem Nobelpreis für Ökonomie ausgezeichnet wurde. Nach der Theorie der ökonomischen Geografie können Steuern als ein Preis für die Attraktivität einer wirtschaftlichen Region interpretiert werden (Baldwin und Krugman, 2004; Hühnerbein und Seidel, 2010). Räume und Volkswirtschaften mit einer hohen Standortattraktivität können dementsprechend hohe Steuern am Markt für Investitionen durchsetzen, während wirtschaftlich weniger attraktive Regionen sich nur geringfügige Steuerbelastungen leisten können. Eine derartige Theorie entspricht dem empirischen Befund und lässt sich zudem durch eine Reihe einfacher Beobachtungen rechtfertigen:

- Für die deutsche Gewerbesteuer erheben wirtschaftlich attraktive Großstädte deutlich höhere Hebesätze als weniger attraktive Gemeinden im ländlichen Raum. Besonders hohe Hebesätze wenden regionale Ballungszentren wie München (490 %), Hamburg (470 %) oder Frankfurt a. M. (460 %) an.[1]

- Große wirtschaftlich attraktive Staaten erheben in aller Regel deutlich höhere Unternehmensteuern als kleinere mit kleineren Märkten und einer geringeren Standortattraktivität. Im Jahr 2019 etwa lag die Steuerbelastung von großen attraktiven Staaten wie Deutschland (29,9 %), Frankreich (32 %), Italien (27,8 %) oder den USA (Staat New York, 27,5 %) deutlich über derjenigen in kleineren oder osteuropäischen Staaten wie Bulgarien (10 %), Litauen (15 %) Polen (19 %), Rumänien (16 %) oder Tschechien (19 %) (Ernst & Young, 2019; OECD.Stat, 2020).

- Als Staat mit attraktiven wirtschaftlichen Regionen (Kalifornien, New York) konnten sich die USA über Jahre hinweg die höchsten Unternehmensteuern in der westlichen Welt leisten, ohne dass dies Wachstum oder wirtschaftlicher Bedeutung erkennbar Abbruch getan hätte. Bemerkenswert ist zudem, dass gerade als mobil geltende Unternehmen der digitalen Wirtschaft (Apple, Google, Facebook) ihren Firmensitz im Staat Kalifornien haben, einer Region, die in den letzten 15 Jahren eine der höchsten Unternehmensteuern weltweit hatte.[2]

### Steuerwettbewerb mit den USA und Europa

Zunächst lässt sich festhalten, dass die nominale Steuerbelastung der meisten wirtschaftlich attraktiven Bundesstaaten sich einschließlich der State Corporate Income Taxes nicht allzu dramatisch vom deutschen Referenzwert von 29,83 % unterscheidet. Mit der State Corporate Income Taxes ergeben sich Gesamtbelastungen von 27,5 % für den Staat New York und 29,84 % für Kalifornien.[3] Hinzu können noch lokale Steuern (etwa 8,85 % für New York City (Bundesfinanzministerium, 2019, 17)) und vermögensbezogene Steuern kommen, die in Deutschland nicht anfallen.

Neben der reinen Senkung der Unternehmensteuersätze enthält der „Tax Cuts and Jobs Act“ noch steuerliche Sonderabschreibungen für Realinvestitionen und begünstigte Steuersätze für Einkünfte aus immateriellen Wirtschaftsgütern, die die effektive Steuerbelastung in den USA weiter absenken. Daher argumentiert die Literatur zum Teil mit effektiven Steuerbelastungen von unter 25 % (Haas und Wünnemann, 2018).[4] Diesen entlastenden Effekten der Steuerreform stehen allerdings auch belastende Faktoren gegenüber, die die Bemessungsgrundlage weit über den in den USA erzielten Gewinn hinaus erweitern können und damit vergleichbar zu gewerbesteuerlichen Hinzurechnungen eine Besteuerung erfolgsunabhängiger Ertragsbestandteile implizieren. So stellt die Base Erosion and Anti-Abuse Tax (BEAT) faktisch eine alternative Mindestbesteuerung dar, die die steuerliche Abzugsfähigkeit von Betriebsausgaben mit Auslandsbezug unter Umständen erheblich einschränkt. Zudem bedeutet die Besteuerung des Global Intangible Low-Taxed Income eine erhebliche Ausweitung der Hinzurechnungsbesteuerung für Einkünfte aus immateriellen Wirtschaftsgütern.[5] Während die Sonderabschreibungen im Jahr 2022 auslaufen, verschärft sich die durch BEAT anfallende Belastung sukzessiv bis zum Jahr 2026.

Damit erscheint zumindest zweifelhaft, dass sich die US-Steuerreform negativ auf die gesamtwirtschaftliche Entwicklung und die Investitionstätigkeit in Deutschland auswirken wird. Insbesondere stellt sich die Frage, welche Relevanz der Besteuerung als Standortfaktor bei der Frage zukommt, ob europäische Unternehmen Produktions- und Forschungskapazitäten in den USA aufbauen oder gar den Unternehmenssitz verlagern. Befragungsdaten sprechen dafür, dass gerade für langfristige strategische Entscheidungen Standortfaktoren wie die Verfügbarkeit von Arbeitskräften, Zuliefernetzwerke, Kooperationsmöglichkeiten mit Forschungseinrichtungen, Arbeitskosten oder die Infrastruktur bedeutsamer sind. Auch scheint unklar, wie nachhaltig die Politik der derzeitigen US-Regierung ausgelegt ist. Die Steuerreform wurde gegen den Widerstand der Demokraten regelrecht durch die Gremien gepeitscht und führte bereits in Zeiten der Hochkonjunktur zu einem massiven Anstieg der Staatsverschuldung. Langfristig dürfte sich diese Position fiskalpolitisch kaum halten lassen. Die in Teilen erratische Politik der Trump-Regierung sollte nicht unbedingt dazu beitragen, die USA als Wirtschaftsstandort attraktiver zu machen.

Im europäischen Vergleich stellt Deutschland demgegenüber inzwischen durchaus ein Hochsteuerland dar. Belgien (25 %), Italien (27,8 %), Griechenland (28 %) und Großbritannien (19 %) haben ihre Steuerbelastungen bereits ge-

1 Dabei scheint es insbesondere auf die direkte lokale Konkurrenzsituation anzukommen. So finden sich etwa auch hohe Hebesätze in ostdeutschen regionalen Ballungskernen wie Magdeburg (450 %), Halle (450 %), Leipzig (460 %) und Dresden (450 %).

2 Vor der US-Steuerreform kam zu der Federal Income Tax von 35 % noch eine State Income Tax von 8,84 % sowie etwaige lokale Steuern. Die Gesamtbelastung betrug somit über 40 % und lag damit eindeutig international in der Spitze bei den Steuerbelastungen von Unternehmen (hierzu auch Bundesfinanzministerium, 2019, 16-17).

3 Die State Corporate Income Taxes der betreffenden Staaten betragen 6,5 % für New York, 8,84 % für Kalifornien. Für andere Staaten ergeben sich zum Teil noch höhere Belastungen. So beträgt etwa die Corporate State Income Tax in Pennsylvania 9,99 %. In 23 Bundesstaaten ergeben sich höhere Belastungen als in New York (Tax Foundation, 2020).

4 Heinemann et al. (2017) ermitteln in einer ersten Analyse der US-Steuerreform unter Berücksichtigung von Abschreibungsbegünstigungen einen effektiven Durchschnittssteuersatz von 22,7 % (Heinemann et al., 2017, 1-5).

5 Eine detailliertere Darstellung dieser Regelungen findet sich etwa bei Linn (2018) oder Schreiber et al. (2018).

senkt und liegen unter der üblicherweise als Referenzwert unterstellten deutschen Steuerbelastung von 29,8 %. Die Situation wird sich voraussichtlich noch dadurch verschärfen, dass einige europäische Staaten weitere steuerliche Entlastungen von Unternehmen planen (Schreiber et al., 2018, 242-243). Dies lässt eine Senkung der Unternehmensteuerbelastung in Deutschland zunächst als naheliegend erscheinen.

Allerdings sollte bei diesen Überlegungen auch die Wettbewerbsposition Deutschlands in der Europäischen Union berücksichtigt werden. Seit der Wiedervereinigung ist Deutschland die größte und wichtigste Volkswirtschaft der EU mit einer hohen Finanzstabilität. Deutschland hat sich in den letzten zehn Jahren deutlich dynamischer entwickelt als Italien oder Frankreich. Vor diesem Hintergrund erscheint es im Sinne der ökonomischen Geografie von Krugman durchaus gerechtfertigt, dass Deutschland für seinen attraktiven Investitionsstandort[6] einen höheren Preis fordert als seine europäischen Wettbewerber. Dass wirtschaftlich schwächere und weniger attraktive Staaten geringere Unternehmensteuern erheben, sollte Deutschland nicht verunsichern. Eine Sondersituation dürfte im Vergleich zu Großbritannien bestehen. Aus der Perspektive der ökonomischen Geografie spiegelt die dramatische Senkung der Unternehmensteuerbelastung auf 19 % die stark gesunkene Attraktivität Großbritanniens infolge des Brexit wider (Zeit, 2019). Ein wesentlicher Gesichtspunkt für die deutsche Steuerpolitik sollte zudem der Einfluss sein, den Deutschland auf die europäischen Nachbarn ausübt. Als ökonomisches Schwergewicht auf dem europäischen Kontinent hat die deutsche Steuerpolitik auch für seine Nachbarn Bedeutung. Wenn sich Deutschland am aktuellen Unterbietungswettbewerb aktiv beteiligt, dann dürfte dies andere europäische Staaten wieder unter Zugzwang setzen.

## Verteilung von Einkommen und Steuerbelastungen

Während Anfang der 1950er Jahre noch etwa 17 % des Gesamtsteueraufkommens auf Unternehmensteuern (Gewerbesteuer, Körperschaftsteuer) und etwa 6 % auf Steuern auf Vermögen und Vermögenstransfers (z. B. Grundsteuer, Vermögensteuer, Erbschaftsteuer, Grunderwerbsteuer) entfielen, waren es in den Jahren 2013 bis 2016 noch etwa 10 % (Unternehmensteuern) bzw. 4 %.[7] Im gleichen Zeitraum ist zudem die Bedeutung von privaten Vermögen im Verhältnis zum Volkseinkommen deutlich gestiegen.[8] Betrug das private Vermögen im Jahr 1950 noch weniger als das Zweifache des Bruttoinlandsprodukts, so hat sich dieses Verhältnis bis zum Jahr 2010 auf mehr als das Vierfache mehr als verdoppelt bei weiterhin steigender Tendenz (Piketty und Zucman, 2014, 1255). Bach (2018) findet Belege für eine steigende Steuerbelastung auf Konsum und Arbeitseinkommen, während der Anteil der Steuern auf Vermögen und Unternehmensgewinne am Gesamtsteueraufkommen zurückgeht.

Gleichzeitig zeigen weithin beachtete Forschungsergebnisse von Thomas Piketty und weiteren Ökonomen, dass die Konzentration von Vermögen und Einkommen in der Spitze der Bevölkerung in Deutschland wie auch in der gesamten westlichen Welt in den letzten Jahrzehnten stark angestiegen ist (Piketty, 2020). Nach Berechnungen von Bartels (2019) stieg zwischen 1961 und 2014 der Anteil der einkommensstärksten 10 % am Volkseinkommen von etwa 30 % auf über 40 %, während gleichzeitig der Anteil der einkommensschwächsten 50 % von etwa 32 % auf 15 % gefallen ist, wie auch in Abbildung 2 zu erkennen ist (Bartels, 2019, 686). Gerade seit Mitte der 1990er Jahre ist eine verstärkte Polarisierung von Einkünften zu beobachten (Bartels, 2018).

Eine derartige Entwicklung lässt sich nur schwer mit Vorstellungen von Verteilungsgerechtigkeit (Hey, 2017, 633) oder dem Leistungsfähigkeitsprinzip in Einklang bringen. Demzufolge sollten gerade die finanziell leistungsfähigen Bevölkerungsschichten, die letztlich identisch mit Vermögenden und insbesondere den Anteilseignern von Unternehmen sind (Bartels, 2019), stärker zur Finanzierung des Steueraufkommens beitragen, als finanziell weniger leistungsfähige „arme“ Bevölkerungsschichten. Dies spricht zudem für eine fortschreitende Spaltung und Entsolidarisierung der deutschen Gesellschaft (Hurst, 2018; Klein, 2018), die derzeit die gesellschaftliche Debatte bestimmt und sich anscheinend auch im Steuersystem niederschlägt. Die derzeit diskutierten Pläne für eine weitere Senkung der Steuerbelastung von Unternehmen drohen diese Tendenzen weiter zu verschärfen.

## Steuerpolitik im Rahmen der COVID-19-Pandemie

Die bisherigen Maßnahmen des COVID-19-Konjunkturpakets sehen keine dauerhaften Senkungen der Steuersätze

6 Laut dem European Attractiveness Survey 2020 liegt Deutschland europaweit auf Platz 3 der attraktivsten wirtschaftlichen Regionen in Bezug auf Direktinvestitionen (Ernst & Young, 2020).

7 Eigene Berechnungen auf Basis der kassenmäßigen Steuereinnahmen nach Steuerarten von 1950 bis 2017 (Bundesfinanzministerium, 2018; Statistisches Bundesamt, 2019, 281).

8 Diese Überlegung gilt allerdings nicht für die Unternehmengewinne. Nach der Volkswirtschaftlichen Gesamtrechnung ist der Anteil des Volkseinkommens aus Unternehmertätigkeit und Vermögen von 1950-1953 bis 2013-2016 von etwa 42 % auf 32 % zurückgegangen. Allerdings erklärt dies nur etwa zur Hälfte den Rückgang des Anteils der Unternehmensteuern am deutschen Steueraufkommen von etwa 17 % auf 10 % im gleichen Zeitraum (vgl. hierzu Statistisches Bundesamt, 2019, 281).

Abbildung 2
**Anteile der Einkommensgruppen am Volkseinkommen**
in %

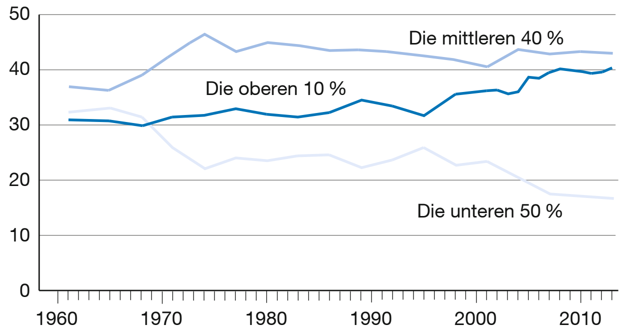

Quelle: Bartels, 2018, 56.

für Unternehmen (Gewerbesteuer, Körperschaftsteuer) vor (vgl. Beck Online, 2020a, 2020b; Korn, 2020). Neben Maßnahmen zur Ankurbelung des Konsums (zeitlich befristete Senkung der Mehrwertsteuer, Kinderbonus von 300 Euro, Senkung der EEG-Umlage, temporäre Erhöhung des Entlastungsbetrags für Alleinerziehende bei der Einkommensteuer), der Förderung der Elektromobilität (Kaufprämien für Elektrofahrzeuge, Erhöhung des maximalen Bruttolistenpreises für eine begünstigte Besteuerung der privaten Nutzung von Elektrofahrzeugen auf 60.000 Euro, Ausbau von Ladenetzen) sind insbesondere auch ertragsteuerliche Erleichterungen zur Schonung der Liquidität sowie steuerliche Anreize für Investitionen vorgesehen.

Um die Liquidität von Unternehmen zu schonen, soll der Verlustrücktrag für die Jahre 2020 und 2021 auf 5 Mio. Euro (bei gemeinsamer Veranlagung von Ehegatten 10 Mio. Euro) angehoben werden. Zudem werden die Fristen für die Verwendung von Investitionsabzugsbeträgen gemäß § 7g sowie die Reinvestitionsfristen für Rücklagen gemäß § 6b EStG temporär um ein Jahr verlängert. Um Investitionsanreize für Unternehmen zu verstärken, wurden weiterhin degressive Abschreibungen von maximal 25 % für Investitionen in den Jahren 2020 und 2021 sowie eine Erhöhung der Forschungszulage von 2 Mio. Euro auf 4 Mio. Euro umgesetzt. Dauerhafte steuerliche Entlastungen ergeben sich bei der Gewerbesteuer. Der Freibetrag für Hinzurechnungen gemäß § 8 Nr. 1 Gewerbesteuergesetz wurde auf 200.000 Euro erhöht, was auch Inflationseffekte der Vorjahre ausgleichen soll (der Freibetrag wurde seit 2008 nicht angepasst). Zudem wurde als Reaktion auf die gestiegenen Hebesätze der Gewerbesteuer der Anrechnungsfaktor gemäß § 35 Einkommensteuergesetz auf maximal 4 erhöht, was das Risiko von steuerlichen Doppelbelastungen für Einzelunternehmen und Personengesellschaften mindert. Diese Maßnahmen erscheinen als durchaus sachgerecht, um einerseits Liquiditätsprobleme in der COVID-19-Pandemie zu vermindern und andererseits Investitionsanreize im Sinne einer antizyklischen Konjunkturpolitik zu erzeugen. Im Verhältnis zu steuerlichen Investitionsanreizen aus früheren konjunkturellen Krisen in den USA wäre gleichwohl zu überlegen, ob der Gesetzgeber nicht noch stärker auf beschleunigte Abschreibungsregelungen setzen sollte, um antizyklische Investitionsanreize zu verstärken.

Sowohl in der Finanzkrise als auch im „Tax Cuts and Jobs Act" haben die USA stark auf steuerliche Sonderabschreibungen (Bonus Depreciation) zurückgegriffen, um den Anreiz für kurzfristige Investitionen zu erhöhen. Derartige Förderinstrumente sind für den Staat bei den aktuellen Zinssätzen vergleichsweise günstig, da Abschreibungen nur beschleunigt werden (etwa 50 % Sonderabschreibung im Jahr der Investition, die weiteren 50 % der Investitionssumme werden über die Nutzungsdauer abgeschrieben). Die Unternehmen sparen aufgrund erhöhter Abschreibungen in der aktuellen Periode zwar Steuern, vermindern damit aber gleichzeitig ihr Abschreibungspotenzial für künftige Perioden. Letztlich führt dies zu einer Verlagerung von Steueraufkommen in die Zukunft, was für den Staat relativ unproblematisch ist, solange die Zinssätze für Staatsanleihen nahe null oder sogar negativ sind. Zugleich entlastet eine solche Politik aber Unternehmen, deren Kapitalkosten deutlich höher liegen.

Bisherige empirische Untersuchungen machen deutlich, dass derartige Sonderabschreibungen, die zeitlich befristet sind, einen wesentlich stärkeren Einfluss auf die Investitionstätigkeit von Unternehmen haben, als dauerhafte Senkungen der Steuersätze von Unternehmen. Dies ist darauf zurückzuführen, dass starke Vorverlagerungseffekte von Investitionen zu erwarten sind und nur Unternehmen von der Förderung profitieren, die auch wirklich in der Krise investieren. Bei zeitlich befristeten Sonderabschreibungen liegen die geschätzten Elastizitäten der Investitionstätigkeit im Verhältnis zu steuerlichen Förderung zwischen 6 und 14 (Eichfelder und Schneider, 2018, 24-25; House und Shapiro, 2008; Maffini et al., 2019; Ohrn, 2018; Zwick und Mahon, 2017), bei dauerhaften Anpassungen der Steuersätze oder der Kapitalnutzungskosten aber nur zwischen 0,25 und 1 (Auerbach und Hassett, 1992; Bond und Xing, 2015; Chirinko et al., 1999; Cummins et al., 1994). Dementsprechend wären zeitlich befristete steuerliche Sonderabschreibungen für Bereiche mit Nachholbedarf (etwa die digitale Infrastruktur) zur Förderung von Investitionen deutlich besser geeignet als eine dauerhafte Senkung der Steuerlast.

Eine Studie des Instituts für Makroökonomie und Konjunkturpolitik (IMK) und des Instituts der deutschen Wirtschaft (iw) macht weiterhin deutlich, dass die öffentliche Hand in den letzten beiden Jahrzehnten Investitionen in den öffentlichen Kapitalstock massiv vernachlässigt hat. Dies umfasst insbesondere Bereiche wie Bildung, Verkehr, Kommunikationsnetze und Dekarbonisierung. Der damit in Zusammenhang stehende Investitionsbedarf wird für die nächsten zehn Jahre auf 450 Mrd. Euro geschätzt (Bardt et al., 2019).

## Fazit

Politiker, Wissenschaftler und Wirtschaftsvertreter fordern immer wieder eine sinkende Steuerbelastung für deutsche Unternehmen und begründen dies mit der Wettbewerbsfähigkeit des Standortes Deutschland. Angesichts dessen nach wie vor hoher Attraktivität erscheinen derartige Maßnahmen aber nicht zweckmäßig. Insbesondere wäre eine Senkung der Unternehmensteuern nicht geeignet, um die Liquidität von Unternehmen in der aktuellen Krisensituation sicherzustellen, da zahlungsunfähige Unternehmen keine Gewinne erwirtschaften. Eine (dauerhafte) Senkung der Unternehmensteuersätze wäre zudem wenig geeignet, um die dringend benötigten antizyklischen Investitionsanreize für Unternehmen zu erzeugen. Zielführender erscheinen demgegenüber zeitlich befristete Maßnahmen zur Erhöhung von Investitionsanreizen, wie Sonderabschreibungen oder Forschungszulagen, die bereits teilweise durch das COVID-19-Konjunkturpaket auf den Weg gebracht wurden. Idealerweise sollten sich derartige steuerliche Maßnahmen auf Sektoren und Bereiche konzentrieren, bei denen ein hoher Investitionsbedarf besteht (etwa digitale Infrastruktur, digitale Prozesse, Dekarbonisierung der deutschen Industrie, Infrastruktur für Elektromobilität).

Die bisherige Debatte negiert leider häufig, dass die Unternehmensbesteuerung keinesfalls den wichtigsten Faktor bei Standortwahlentscheidungen darstellt, sondern sich bei Befragungen üblicherweise als ein mittelmäßig relevanter Standortfaktor erweist. Vernachlässigt wird zudem, dass die (digitale) öffentliche Infrastruktur, die Digitalisierung der Gesellschaft und die Nachhaltigkeit der Energieerzeugung Standortfaktoren sind, die deutlich wichtiger sind als die ertragsteuerliche Belastung von Unternehmen (EY, 2020; Weltbank, 2020). Demzufolge wäre es unter dem Gesichtspunkt der Standortattraktivität Deutschlands vermutlich zielführender, bestehende steuerliche Vergünstigungen für privilegierte Schichten abzubauen (etwa die steuerliche Nichterfassung von Spekulationsgewinnen im Privatvermögen oder die massiven Begünstigungen für Betriebsvermögen bei der Erbschaftsteuer, Eichfelder, 2018, 2400 ff.) und gleichzeitig die öffentliche Investitionstätigkeit in die erläuterten Bereiche massiv zu erhöhen, um den identifizierten Investitionsstau für öffentliche Investitionen abzubauen.

Eine derartige Politik würde aller Wahrscheinlichkeit nach die Standortattraktivität Deutschlands erhöhen und zugleich die massive und zunehmende Ungleichheit in der Verteilung von Einkommen und Vermögen vermindern. Dabei ist zu berücksichtigen, dass Anteilseigner von Unternehmen und Vermögende bereits stark von den steuerlichen Reformbemühungen der letzten 20 Jahre profitiert haben und zugleich ökonomische Gewinner der Globalisierung sind. Profiteure einer neuen Unternehmensteuerreform wären also genau diejenigen Akteure, die ihre ökonomischen Positionen in den letzten Jahren ohnehin stark verbessern konnten. Eine Steuerpolitik, die derartige Zusammenhänge und Probleme ignoriert, riskiert dauerhaft den gesellschaftlichen Zusammenhalt, der vermutlich einen der wichtigsten Faktoren für den Wirtschaftsstandort Deutschland darstellt.

**Title:** ***Tax Policy in the COVID-19 Crisis***

**JEL Classification:** *H25, H71*

## References

[CR1] Auerbach A J, Hassett K A (1992). Tax policy and business fixed investment in the United States. Journal of Public Economics.

[CR2] Bach, S. (2018), 100 Jahre deutsches Steuersystem: Revolution und Evolution, Arbeitspaper, DIW Berlin.

[CR3] Baldwin R E, Krugman P (2004). Agglomeration, integration and tax harmonisation. European Economic Review.

[CR4] Bardt, H., S. Dullien, M. Hüther und K. Rietzler (2019), Für eine solide Finanzpolitik: Investitionen ermöglichen, *IMK Report*, 152.

[CR5] Bartels C (2018). Einkommensverteilung in Deutschland von 1871 bis 2013: Erneut steigende Polarisierung seit der Wiedervereinigung. DIW Wochenbericht.

[CR6] Bartels C (2019). Top incomes in Germany, 1871–2014. Journal of Economic History.

[CR7] Beck Online (2020a), Corona-Konjunkturpaket: Welche Hilfen für wen geplant sind, Beck Online Redaktion beck-aktuell, 4. Juni, *becklink 2016506*, https://rsw.beck.de/aktuell/daily/meldung/detail/corona-konjunkturpaket-welche-hilfen-fuer-wen-geplant-sind (24. Juni 2020).

[CR8] Beck Online (2020b), Bundesregierung gibt grünes Licht für Corona-Konjunkturpaket, Beck Online Redaktion beck-aktuell, 12. Juni, *becklink 2016574*, https://rsw.beck.de/aktuell/daily/meldung/detail/bundes-regierung-gibt-gruenes-licht-fuer-corona-konjunkturpaket (24. Juni 2020).

[CR9] Börse Online (2020), Söder fordert wegen Corona-Krise Steuersenkungen und Steuerreform, https://www.boerse-online.de/nachrichten/aktien/soeder-fordert-wegen-corona-krise-steuersenkungen-und-steuerreform-1029624602 (26. September 2020).

[CR10] Bond S, Xing J (2015). Corporate taxation and capital accumulation: Evidence from sectoral panel data for 14 OECD countries. Journal of Public Economics.

[CR11] Bundesfinanzministerium (2018), Kassenmäßige Steuereinnahmen nach Steuerarten 1950 bis 2017, https://www.bundesfinanzministerium.de/Content/DE/Standardartikel/Themen/Steuern/Steuerschaetzungen_und_Steuereinnahmen/2-kassenmaessige-steuereinnahmen-nach-steuerarten-1950-bis-2017.html (3. Februar 2020).

[CR12] Bundesfinanzministerium (2019), Die wichtigsten Steuern im internationalen Vergleich 2018, https://www.bundesfinanzministerium.de/Content/DE/Downloads/Broschueren_Bestellservice/2019-08-08-die-wichtigsten-steuern-im-internationalen-vergleich-2018-ausga-be-2019.pdf?__blob=publicationFile&v=9 (20. Januar 2020).

[CR13] Bundesministerium für Wirtschaft und Energie (2019), Altmeier benennt vier Kernelemente einer Unternehmensteuerreform, https://www.bmwi.de/Redaktion/DE/Pressemitteilungen/2019/20191115-altmaier-benenntkernelemente-unternehmenssteuerreform.html (20. Januar 2020).

[CR14] CDU, CSU und SPD (2018), Koalitionsvertrag 2018, https://www.bundes-regierung.de/resource/blob/656734/847984/5b8bc23590d4cb2892b31c987ad672b7/2018-03-14-koalitionsvertrag-data.pdf?download=1 (20. Juni 2020).

[CR15] CDU/CSU-Fraktion (2019), Positionspapier „Modernisierung der Unternehmenbesteuerung in Deutschland“ der CDU/CSU-Fraktion im Deutschen Bundestag, https://www.cducsu.de/sites/default/files/2019-11/Positionspapier%20zur%20Modernisierung%20der%20Unternehmenbesteuerung_17102019….pdf (22. Januar 2020).

[CR16] Chirinko R S, Fazzari und S M, Meyer A P (1999). How responsive is business capital formation to its user cost? An exploration with micro data. Journal of Public Economics.

[CR17] Cummins J G, Hassett und K A, Hubbard R G (1994). A reconsideration of investment behavior using tax reforms as natural experiments. Brookings Papers of Economic Activity.

[CR18] Ebertz, A., M. Kriese, M. Thum und E. Seitz (2008), Bewertung von lokalen Standortfaktoren für Haushalte und Unternehmen in Sachsen: Entwicklung von Indikatoren zur Überprüfung der Demographietauglichkeit von Förderprojekten der Sächsischen Aufbaubank: Gutachten im Auftrag der Sächsischen Aufbaubank, *Ifo-Dresden-Studien*, 46.

[CR19] Eichfelder S (2008). Teilsteuerrechnung nach der Unternehmensteuerreform 2008/2009. Der Steuerberater.

[CR20] Eichfelder S (2018). Braucht Deutschland eine neue Unternehmensteuerreform?. Deutsches Steuerrecht.

[CR21] Eichfelder, S. und K. Schneider (2018), How do tax incentives affect business investment: Evidence from German bonus depreciation, *arqus Working Paper*, 231.

[CR22] Ernst & Young (EY, 2019), EY worldwide corporate tax guide 2019, https://assets.ey.com/content/dam/ey-sites/ey-com/en_gl/topics/tax/hc-alert/ey-worldwide-corporate-tax-guide-2019.pdf (24. Juni 2020).

[CR23] EY (2020), EY Attractiveness Survey Europe 2020, https://assets.ey.com/content/dam/ey-sites/ey-com/en_gl/topics/attractiveness/ey-europe-attractiveness-survey-2020-v3.pdf (24. Juni 2020).

[CR24] Feld L P, Heckemeyer J H (2011). FDI and taxation: A meta-study. Journal of Economic Surveys.

[CR25] Fuest C, Peichl A (2020). Acht Elemente einer grundlegenden Reform des Steuer- und Transfersystems. Wirtschaftsdienst.

[CR26] Haas W, Wünnemann M (2018). Steuerpolitik in der 19. Legislaturperiode: Globaler Steuerwettbewerb ist Anlass für notwendige Strukturreformen. Deutsches Steuerrecht.

[CR27] Hebous S, Ruf und M, Weichenrieder A (2011). The effects of taxation on the location decisions of multinational firms: M&A versus greenfield investments. National Tax Journal.

[CR28] Heinemann, F., O. Pfeiffer, T. Schwab, C. Spengel, M. Olbert und K. Stutzenberger (2017), Analysis of US corporate tax reform proposals and their effects for Europe and Germany, *ZEW-Gutachten und Forschungsberichte.*

[CR29] Hey J (2017). Steuerrecht zwischen Umverteilung und Standortpolitik. Steuerpolitische Aufgaben der 19. Legislaturperiode. Deutsche Steuer-Zeitung.

[CR30] Homburg S (2020). Steuersenkungen — Wenn nicht jetzt wann dann?. Wirtschaftsdienst.

[CR31] House C L, Shapiro M D (2008). Temporary investment tax incentives: Theory with evidence from bonus depreciation. American Economic Review.

[CR32] Hühnerbein O, Seidel T (2010). Intra-regional tax competition and economic geography. The World Economy.

[CR33] Hurst, F. (2018), Studie zu sozialer Ungleichheit: Hier arm, dort reich, *Tagesschau*, 23. Mai, https://www.tagesschau.de/inland/soziale-ungleichheit-101.html (22. Juni 2020).

[CR34] Hüther M (2020). Zeit für Wachstumspolitik. Wirtschaftsdienst.

[CR35] Kimelberg S M, Williams E (2013). Evaluating the importance of business location factors: The influence of facility type. Growth and Change.

[CR36] Klein, J. (2018), Soziale Durchmischung: Die Spaltung wird größer, *Frankfurter Allgemeine Zeitung*, 2. Juni, https://www.faz.net/aktuell/politik/inland/interview-mit-sozialforscher-die-spaltung-wird-groesser-15618171.html (22. Juni 2020).

[CR37] Korn C (2020). Das zweite Corona-Steuerhilfegesetz im Überblick. Deutsches Steuerrecht.

[CR38] Landua, D., S. Wagner-Endres und U. Wolf (2017), Kurzstudie zu kommunalen Standortfaktoren: Ergebnisse auf Grundlage der Daten des Difu-Projekts „Koordinierte Unternehmenbefragung“, KfW Research.

[CR39] Linn A (2018). Die US-Unternehmensteuerreform und ihre Auswirkungen auf das deutsche Unternehmensteuerrecht. Deutsches Steuerrecht.

[CR40] Linnemann N, Weiß F (2019). Braucht Deutschland doch (k)eine Unternehmensteuerreform?. Internationales Steuerrecht.

[CR41] MacCarthy B L, Atthirawong W (2003). Factors affecting location decisions in international operations — A Delphi study. International Journal of Operations & Production Management.

[CR42] Maffini G, Xing und J, Devereux M (2019). The impact of investment incentives: Evidence from UK corporation returns. American Economic Journal: Economic Policy.

[CR43] OECD (2019), Tax policy reforms 2019, https://www.oecd.org/tax/tax-policy-reforms-26173433.htm (8. Januar 2020).

[CR44] OECD.Stat (2020), Tax database, Table https://stats.oecd.org/Index.aspx?QueryId=78166 (24. Juni 2020).

[CR45] Ohrn E (2018). The effect of corporate taxation on investment and financial policy: Evidence from the DPAD. American Economic Journal: Economic Policy.

[CR46] Piketty, T. (2020), *Das Kapital im 21. Jahrhundert*, Beck.

[CR47] Piketty T, Zucman G (2014). Capital is back: Wealth-income ratios in rich countries. Quarterly Journal of Economics.

[CR48] Schreiber U, von Hagen und D, Pönnighaus F N (2018). Nach der US-Steuerreform 2018: Deutschland im Steuerwettbewerb. Steuer und Wirtschaft.

[CR49] Statistisches Bundesamt (2019), *Statistisches Jahrbuch 2019.*

[CR50] Tax Foundation (2020), State Corporate Income Tax tates and brackets for 2020, https://taxfoundation.org/state-corporate-income-tax-rates-brackets-2020/ (14. Februar 2020).

[CR51] Vlachou C, Iakovidou O (2015). The evolution of studies in business location factors. Journal of Developmental Entrepreneurship.

[CR52] Welling B (2018). Notwendigkeit der steuerlichen Strukturreformen. Der Betrieb.

[CR53] Weltbank (2020), Ease of Doing Business rankings, https://www.doing-business.org/content/dam/doingBusiness/pdf/db2020/Doing-Business-2020_rankings.pdf (24. Juni 2020).

[CR54] Wissenschaftlicher Beirat beim Bundesministerium der Finanzen (2018), US-Steuerreform 2018: Steuerpolitische Folgerungen für Deutschland, https://www.bundesfinanzministerium.de/Content/DE/Down-loads/Broschueren_Bestellservice/2019-03-15-WB-US-Steuerre-form-2018.pdf?__blob=publicationFile&v=6 (20. Februar 2020).

[CR55] Wolff, G. (2018), Die Folgen der US-Steuerreform für Europa: Warum die Bundesregierung auf Trumps Reform rasch reagieren muss, *Manager-Magazin*, 14. Februar.

[CR56] Zeit (2019), Statistikamt: Britische Wirtschaft schrumpft erstmals seit 2012, https://www.zeit.de/news/2019-08/09/britische-wirtschaft-schrumpft-erstmals-seit-dem-jahr-2012 (14. Februar 2020).

[CR57] Zwick E, Mahon J (2017). Tax policy and heterogenous investment behavior. American Economic Review.

